# Vimentin, colon cancer progression and resistance to butyrate and other HDACis

**DOI:** 10.1111/jcmm.12850

**Published:** 2016-04-12

**Authors:** Darina L. Lazarova, Michael Bordonaro

**Affiliations:** ^1^ Department of Basic SciencesThe Commonwealth Medical CollegeScrantonPAUSA

**Keywords:** vimentin, epithelial to mesenchymal transition, microadenoma, colorectal cancer, butyrate, HDACis

## Abstract

Dietary fibre protects against colorectal cancer (CRC) most likely through the activity of its fermentation product, butyrate. Butyrate functions as a histone deacetylase inhibitor (HDACi) that hyperactivates Wnt signalling and induces apoptosis of CRC cells. However, individuals who consume a high‐fibre diet may still develop CRC; therefore, butyrate resistance may develop over time. Furthermore, CRC cells that are resistant to butyrate are cross‐resistant to clinically relevant therapeutic HDACis, suggesting that the development of butyrate resistance *in vivo* can result in HDACi‐resistant CRCs. Butyrate/HDACi‐resistant CRC cells differ from their butyrate/HDACi‐sensitive counterparts in the expression of many genes, including the gene encoding *vimentin* (*VIM*) that is usually expressed in normal mesenchymal cells and is involved in cancer metastasis. Interestingly, vimentin is overexpressed in butyrate/HDACi‐resistant CRC cells although Wnt signalling is suppressed in such cells and that *VIM* is a Wnt activity‐targeted gene. The expression of vimentin in colonic neoplastic cells could be correlated with the stage of neoplastic progression. For example, comparative analyses of LT97 microadenoma cells and SW620 colon carcinoma cells revealed that although vimentin is not detectable in LT97 cells, it is highly expressed in SW620 cells. Based upon these observations, we propose that the differential expression of vimentin contributes to the phenotypic differences between butyrate‐resistant and butyrate‐sensitive CRC cells, as well as to the differences between early‐stage and metastatic colorectal neoplastic cells. We discuss the hypothesis that vimentin is a key factor integrating epithelial to mesenchymal transition, colonic neoplastic progression and resistance to HDACis.

**Table:** 

• Main hypothesis
• Wnt signalling and butyrate
• Vimentin and epithelial to mesenchymal transition in colorectal cancer cells
• Vimentin and cell signalling
• Future directions: hypothesis testing
• Conclusion

## Main hypothesis

We posit that, in neoplastic cells resistant to butyrate, vimentin expression at least in part mediates the association between histone deacetylase inhibitor (HDACi) resistance and epithelial to mesenchymal transition (EMT) by simultaneously influencing (*i* ) cell migration/invasiveness and (*ii*) cell signalling/gene expression that promotes resistance to HDACis.

## Wnt signalling and butyrate

The HDACi butyrate, produced by fermentation of dietary fibre in the colon, hyperactivates Wnt signalling in colorectal cancer (CRC) cells, resulting in enhanced levels of apoptosis and repressed cell growth 1, 2, 3, 4, 5, 6, 7. Therefore, the preventive action of fibre against CRC is at least partially attributed to the ability of butyrate to modulate Wnt signalling and its downstream physiological consequences 1, 2, 3, 4, 5, 6, 7.

Wnt activity results from increased levels of active beta‐catenin, which associates with DNA‐binding Tcf factors to stimulate transcriptional activity from various genes 8, 9, 10, 11. Deregulated Wnt activity may result from mutations in *APC* and *CTNNB1* (encoding beta‐catenin) genes, promoting abnormal colonic cell proliferation and tumorigenesis 8, 9, 10, 11. The physiological consequences of Wnt signalling in colonic cells exists in a continuum: whereas, moderate Wnt activity levels drive proliferation, relatively low or high levels of Wnt signalling activity repress proliferation, and drive apoptosis, of transformed colonic cells 4, 5, 6, 7. Thus, levels of Wnt signalling higher than those induced by *APC* or *CTNNB*1 mutations promote CRC cell apoptosis. Such hyperactivation of Wnt signalling takes place when CRC cells with *APC* or *CTNNB1* mutations are exposed to butyrate; under such conditions, the growth suppressive and apoptotic effects of butyrate are linearly and causatively correlated with the fold up‐regulation of Wnt activity 5, 12. Related to those changes in cell physiology, butyrate influences the expression of a wide range of Wnt activity‐targeted genes in CRC cells 13.

However, individuals with a high‐fibre diet may still develop CRC, and such colonic neoplastic development could be attributed to the acquisition of resistance to the chemopreventive action of butyrate. To evaluate the mechanisms leading to butyrate resistance, we have developed a butyrate‐resistant CRC cell line, HCT‐R, which is also cross‐resistant to clinically relevant therapeutic HDACis; HCT‐R cells were derived from butyrate/HDACi‐sensitive HCT‐116 cells 6. We have previously observed that butyrate‐resistance in HCT‐R cells is partially mediated by enhanced expression of several cell‐cycle factors, and that of the transcription factor Tcf3, which inhibits Wnt/beta‐catenin signalling 14. Thus, a switch from canonical (*i.e*. beta‐catenin‐dependent) to increased non‐canonical (not dependent upon beta‐catenin) Wnt signalling mediates the butyrate‐resistant HCT‐R cell phenotype 15. HCT‐R cells are also deficient in expression of the histone acetylase p300 16, a protein that mediates the effects of Wnt signalling and modulates the ability of butyrate to affect Wnt signalling (16 and refs. therein).

However, these findings do not entirely explain the butyrate‐resistant phenotype of HCT‐R cells; thus, additional genes that influence colonic cell physiology likely contribute to the development of butyrate resistance. In support of this possibility, microarray analyses have revealed that vimentin is overexpressed in butyrate‐resistant HCT‐R cells compared to butyrate‐sensitive parental HCT‐116 CRC cells 14. The expression of vimentin is likely negatively correlated with the expression and activity of p300. Thus, p300 knockout (KO) HCT‐116 lines express higher levels of vimentin, and exhibit greater migration/invasiveness, compared to the parental p300‐expressing HCT‐116 cells 17. Therefore, the increased expression of vimentin in HCT‐R cells may mediate the effects of p300 deficiency on cell physiology, including EMT and the acquisition of resistance to butyrate and other HDACis.

## Vimentin and epithelial to mesenchymal transition in colorectal cancer cells

*VIM*, the gene encoding the intermediate filament protein vimentin involved in cellular structure and integrity, is a Wnt activity‐targeted gene expressed in normal mesenchymal cells. Vimentin influences cell shape and motility in the process of EMT that occurs during metastasis (18, 19, 20, 21, 22, 23 and refs. therein). The importance of vimentin in EMT is underscored by the fact that siRNA knockdown of vimentin in CRC cells reduces cell migration and invasiveness 20. Therefore, it is of interest to evaluate how the expression of vimentin is modulated along (*i*) the neoplastic progression in the colon and (*ii*) the acquisition of a butyrate/HDACi‐resistant phenotype. This question could be addressed by examining *in vitro* models of the progressive neoplastic phenotypes in the colon. Below we discuss two such cell culture‐based models: LT97 microadenoma cells and SW620 colon carcinoma cells.

*In vitro* studies investigating preventive approaches against CRC have typically used carcinoma cells, instead of cells representative of earlier stages of colonic neoplasia (*i.e*. adenoma stage). It is likely that the preventive action of butyrate is most effective at the early stage of colonic neoplasia. Thus, studies on the effects of fibre, the dietary source of butyrate, have reported an inverse association of fibre intake with adenoma risk 1, 2, 3. Consistent with these findings, LT97 microadenoma cells, isolated from the earliest stage colonic neoplasm 23, are more sensitive to the growth suppressive effects of butyrate than colon carcinoma HT‐29 cells 24.

In contrast to LT97 cells, SW620 cells represent an *in vitro* model of CRC progression to metastasis. SW620 cells were derived from a lymph node metastasis of a primary colon tumour; whereas cells from the primary tumour in the same patient were used to establish the SW480 cell line 22. SW620 cells have a fibroblast‐like morphology, unlike SW480 cells which appear more epithelial‐like. Both of these CRC cell lines express vimentin 22; therefore, it is possible that the original CRC from which the SW480 cells were derived was further along the neoplastic spectrum than is typical for other CRCs that express little or no vimentin. Compared to SW480 cells, however, SW620 cells exhibit a more advanced neoplastic phenotype; thus, levels of E‐cadherin were observed to be lower in SW620 compared to SW480 cells 22, consistent with the interpretation that SW620 cells have a more EMT‐like phenotype.

Based upon the understanding that LT97 and SW620 cells represent the very early and late stages of colonic tumorigenesis, respectively, we have performed comparative microarray analyses of these cells to identify differences in basal and butyrate‐modulated gene expression. The screening studies consisted of a single replicate of a full human genome analysis to identify genes of interest, and selected gene targets were subsequently validated by Western blot analyses 25. Among these genes, microarray data revealed that vimentin is highly expressed in SW620 carcinoma cells compared to LT97 microadenoma cells, both in the presence and absence of butyrate, and this finding was validated by Western blotting 25.

## Vimentin and cell signalling

Vimentin is a potential cancer therapeutic target, since it is overexpressed in a number of cancers, and it is linked to metastatic progression 18, 19, 20, 21, 26, 27, 28, 29. Besides its well‐known role in affecting cell structure, integrity, and reaction to stress, vimentin may influence cell signalling. For example, it has been shown that vimentin localizes to the nucleus of neuroblastoma cells and up‐regulates the expression of p21Waf1 through increased *p21* promoter activity 32.

Vimentin also associates with the transcription factor ATF4, interfering with that factor's interaction with the *osteocalcin* gene promoter; therefore, vimentin represses osteocalcin expression and inhibits osteoblast differentiation 33. In addition, vimentin associates with, and stabilizes, the phosphorylated form of the MAP kinase ERK, associates with 14‐3‐3 protein to affect cell signalling and cell‐cycle control, interacts/regulates factors involved in EMT 31 and facilitates transport of kinases into the nucleus 34. Effects of vimentin on nuclear shape and architecture 35 may also indirectly modulate gene expression. Therefore, vimentin has cell signalling, as well as cell‐structural roles in affecting and promoting carcinogenesis and metastasis.

## Future directions: hypothesis testing

**Figure 1:**
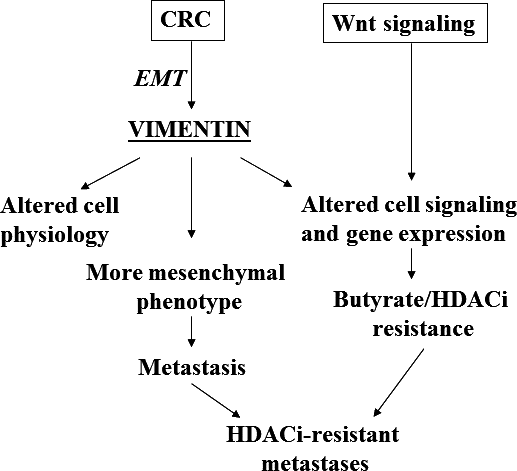
Proposed vimentin interactions in CRC. Deregulated Wnt signalling alters other cell signalling pathways and modulates gene expression, thus promoting CRC;* Vimentin* is a Wnt target gene that is up‐regulated during CRC EMT. Increased levels of vimentin can also modulate cell signalling, possibly altering gene expression, changes in gene expression likely contributes to resistance to butyrate and other HDACis. At the same time, vimentin changes cell physiology, resulting in a more mesenchymal phenotype, promoting metastasis. The combination of EMT and altered gene expression involving response of CRC cells to HDACis can potentially result in HDACi‐resistant metastases. In the figure, arrows represent positive interactions that promote the next step in the neoplastic process.

Our hypothesis that vimentin is a key factor integrating EMT, neoplastic progression and resistance to HDACis in the colon (Fig. 1) is supported by the literature cited above, the strong link between vimentin‐mediated EMT and drug resistance 36, and our gene expression data, according to which butyrate‐resistant CRC cells consistently up‐regulate vimentin within the context of a p300‐deficient, EMT‐like phenotype that exhibits suppressed expression of E‐cadherin (14, 16, unpublished data).

**Figure 2:**
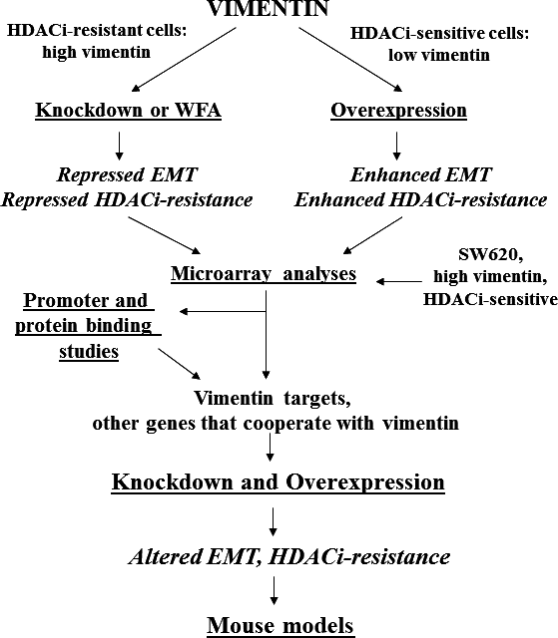
Testing the hypothesis. Some possible approaches to evaluate our hypothesis are diagrammed. Cell culture experiments can start with HDACi‐resistant CRC cells that express higher levels of vimentin (*e.g*. HCT‐R) that will be subjected to vimentin knockdown (or WFA treatment) as well as HDACi‐sensitive, low vimentin‐expressing CRC cells (*e.g*. HCT‐116) subjected to vimentin overexpression. Altered vimentin expression would be expected to change HDACi‐resistance and EMT phenotypes. These cells can be compared to the metastatic SW620 CRC cell line that expresses higher levels of vimentin but is HDACi‐sensitive to identify, by microarray analyses, genes that affect resistance and EMT and that are either targets of vimentin‐mediated cell signalling or that are not vimentin targets but cooperate with vimentin to alter cell phenotype. Promoter (CHiP) and protein (column)‐binding studies can identify novel binding partners and activities for vimentin. Identified genes can be used for further knockdown/overexpression experiments to ascertain how the effects of vimentin on the HDACi‐resistant/EMT phenotype are mediated. These *in vitro* experiments can be followed by *in vivo* studies as described in the text. In the figure, arrows represent the step‐wise approaches proposed to test our hypothesis.

The hypothesis can be tested by vimentin knockdown and overexpression experiments (Fig. 2). Vimentin expression will be suppressed by CRISPR approaches in butyrate‐resistant, p300‐deficient CRC cells (*e.g*. HCT‐R cells) and SW620 metastatic CRC cells; whereas vimentin will be overexpressed in butyrate‐sensitive cells (*e.g*. HCT‐116, LT97 microadenoma cells). A complimentary experimental approach is that of applying the tumour inhibitor and antiangiogenic agent 26 Withaferin‐A (WFA). Withaferin‐A induces vimentin cleavage and apoptosis in vimentin‐expressing cancer cells; however, these effects are markedly less pronounced in normal mesenchymal cells 21, 26, 27, 28, 29. Thus, WFA can be used to down‐regulate vimentin in CRC cells such as SW480 and SW620. The results of this approach should confirm the data obtained by the knockdown of vimentin with CRISPR.

We expect that the knockdown of vimentin in butyrate‐resistant, p300‐deficient HCT‐R cells will not only reverse the EMT‐like phenotype, but, considering the association between EMT and butyrate/HDACi resistance, would at least partially reverse this resistance. Exogenous vimentin overexpression in butyrate‐sensitive CRC cells such as HCT‐116 is expected to promote a more EMT‐like phenotype and allow for increased resistance to the effects of butyrate and HDACis. Resistance to HDACi‐induced cell growth inhibition and apoptosis will be evaluated not only for butyrate but also for clinically relevant therapeutic HDACis such as vorinostat and LBH589. Further, the knockdown of vimentin is expected to at least partially reverse the EMT‐like phenotype observed in the p300‐KO HCT‐116 cells, providing support to the hypothesis that increased vimentin expression in these cells mediates the effects of p300 deficiency on cell physiology.

The interpretation of the experimental data for the metastatic SW620 cells would be more challenging. Thus, these cells exhibit a degree of EMT phenotype and relatively high levels of vimentin; however, they are still moderately sensitive to the cell growth and apoptotic effects of butyrate compared to nine other CRC cell lines 5. In SW620 cells, we expect that additional changes in cell signalling are required before butyrate resistance occurs; therefore, in these cells, increased expression of vimentin is necessary, but not sufficient for the acquisition of butyrate/HDACi‐resistant phenotype.

A comparison of the gene expression profiles of high‐vimentin butyrate‐sensitive SW620 cells to HCT‐R cells ± vimentin knockdown, and HCT‐116 cells ± vimentin overexpression, as well as to LT97 cells, will identify gene expression that cooperates with vimentin in the proposed vimentin‐mediated association between EMT and butyrate/HDACi resistance. The identified differences in gene expression will be correlated with changes in cell phenotype (*e.g*. proliferation, apoptosis, adherent‐independent growth, migration and invasiveness) to ascertain the relative role of these genes in the vimentin‐mediated association between EMT and HDACi resistance. Therefore, full human genome microarray analyses need to be performed on the aforementioned cell lines. Once changes in relevant gene expression are confirmed at the protein level, overexpression and knockdown experiments would be employed to ascertain (*i*) the vimentin‐targeted genes (the expression of which is directly or indirectly modulated by vimentin) that act downstream of vimentin to influence cell phenotype, and (*ii*) the genes that are not vimentin‐targeted genes (*i.e*. genes the expression of which is not modulated by vimentin) that cooperate with vimentin in modulating cell phenotype. One expectation is that, consistent with a role for vimentin in promoting neoplastic progression, vimentin KO SW620 cells would exhibit a gene expression profile more similar to that found in early‐stage LT97 microadenoma cells.

Since the effects of vimentin on gene expression can be direct or indirect, to probe for direct effects, chromatin immunoprecipitation experiments will ascertain whether vimentin associates, directly or indirectly (through transcriptional factors), to the promoter regions of relevant genes whose expression is altered by vimentin. Column chromatography studies using bound vimentin and relevant cell extracts will be used to identify novel binding partners and activities for vimentin that mediate direct or indirect effects of vimentin on gene expression and cell signalling. Thus, novel functions of vimentin in modulating gene expression in CRC, influencing carcinogenesis, neoplastic progression and butyrate resistance, will be ascertained by *in vitro* studies.

The *in vitro* studies will be followed up by *in vivo* studies. For example, vimentin KO mouse models exist, and vimentin KO mice have been shown to be less sensitive to colitis than their wild‐type counterparts 37. The common murine CRC model with a mutation in *Apc* (Apc*min*) exhibits vimentin expression in intestinal adenomas 38; therefore, crossing the vimentin KO and Apc*min* mice is expected to produce hybrid mice that are more sensitive to the tumour‐suppressing effect of butyrate than Apc*min* mice 39. Cell lines derived from intestinal adenomas of vimentin KO‐Apc*min* mice are also expected to show greater sensitivity to HDACis and a less pronounced EMT phenotype, compared to cells derived from Apc*min* adenomas. We also anticipate that exogenous expression of vimentin in the vimentin KO‐Apc*min* cells will (*i*) reverse any observed increase in the HDACi sensitivity and (*ii*) promote a more mesenchymal phenotype.

## Conclusion

Vimentin, through its effects on cancer cellular morphology and cell signalling/gene expression cascades, likely integrates the process of EMT with the acquisition of resistance to butyrate and other HDACis. The result of such activity would be the establishment of metastases of drug‐resistant CRC cells. Therefore, vimentin might be a relevant target for cancer therapeutic interventions. In this paper, we have discussed supporting evidence for our hypothesis and have outlined the experiments that will address the hypothesis.

## Conflict of interest

The authors confirm that there are no conflicts of interest.
